# Toward Exposing Timing-Based Probing Attacks in Web Applications †

**DOI:** 10.3390/s17030464

**Published:** 2017-02-25

**Authors:** Jian Mao, Yue Chen, Futian Shi, Yaoqi Jia, Zhenkai Liang

**Affiliations:** 1 School of Electronic and Information Engineering, Beihang University, 37 Xueyuan Road, Beijing 100191, China; cy229266@163.com (Y.C.); futianbuaa@163.com (F.S.); 2 Department of Computer Science, National University of Singapore, 13 Computing Drive, Singapore 117417, Singapore; jiayaoqi@comp.nus.edu.sg (Y.J.); liangzk@comp.nus.edu.sg (Z.L.)

**Keywords:** side channel, probing attack, web security, privacy

## Abstract

Web applications have become the foundation of many types of systems, ranging from cloud services to Internet of Things (IoT) systems. Due to the large amount of sensitive data processed by web applications, user privacy emerges as a major concern in web security. Existing protection mechanisms in modern browsers, e.g., the same origin policy, prevent the users’ browsing information on one website from being directly accessed by another website. However, web applications executed in the same browser share the same runtime environment. Such shared states provide side channels for malicious websites to indirectly figure out the information of other origins. Timing is a classic side channel and the root cause of many recent attacks, which rely on the variations in the time taken by the systems to process different inputs. In this paper, we propose an approach to expose the timing-based probing attacks in web applications. It monitors the browser behaviors and identifies anomalous timing behaviors to detect browser probing attacks. We have prototyped our system in the Google Chrome browser and evaluated the effectiveness of our approach by using known probing techniques. We have applied our approach on a large number of top Alexa sites and reported the suspicious behavior patterns with corresponding analysis results. Our theoretical analysis illustrates that the effectiveness of the timing-based probing attacks is dramatically limited by our approach.

## 1. Introduction

Web applications have become the foundation of our Internet infrastructure, such as online banking and government services. Recently, it has been used to develop platforms for the Internet of Things (IoT) [1,2,3]. As the web platform gains popularity and attracts a large amount of sensitive data, privacy concerns also emerge. For example, sensitive IoT data can be leaked from web applications or smart devices [4]. In web applications, the same origin policy (SOP) [5] prevents a web application from directly accessing information belonging to other web applications. However, since the web application shares the same browser environment with web applications under other origins, it may indirectly figure out information from other web applications through analyzing the shared states in the browser environment [6,7,8,9,10,11,12]. For example, attackers can infer whether a website has been visited by the user via checking the color of a link to the site, as demonstrated in the browser-history sniffing attack [13]. Using this technique, via including a list of links pointing to a list of websites, a malicious website can obtain a user’s browsing history by checking the color of the links (The traditional way to do this is by calling the getComputedStyle method, but this method has already been modified to prevent this kind of misuse. However, there are still other ways to check the link color [14,15]). This example shows that an attacker can steal users’ information indirectly, i.e., inferring the information about another site by checking the browser states that are affected by visiting the website.

Compared to attacks directly accessing users’ private information in the browser environment, this type of inference attack can only obtain limited information in each attempt. For example, the browser history inference is obtained site-by-site. In other words, the “data rate” of information leakage in such attacks is low. To obtain a significant amount of information, attackers must *repeatedly* check and infer information from the other origin. We call them browser probing attacks and the repetitive nature of browser probing attacks forms the basis to detect them.

We focus on timing-based probing attacks [14,16,17,18] in this paper, which are popular browser probing attacks. They indirectly access sensitive information, such as cryptographic keys and states from other virtual machines [19,20]. They rely on the variations in the time taken by the systems to process different inputs [14,16]. These attacks have been adopted to exploit web applications. In a timing-based probing attack, a malicious web application checks the time required to perform various tasks that can be affected by other websites.

**Our approach:** In this paper, we present a tool to detect timing-based probing attacks in web applications. The main idea of our approach is to monitor a web application’s runtime behaviors and identify anomalous timing operations to detect timing probing attacks. We summarize behavior patterns for timing-based probing attacks, and our approach detects timing probing attacks by matching monitored behaviors with such behavior patterns. Our approach alarms users with the potential risk of the privacy leakage to the website and prompts users with the suspicious behaviors embedded in a malicious web page.

We have prototyped our system in the Google Chrome browser and evaluated the effectiveness of our approach using malicious probing websites [14,16,17,18].

**Contributions.** We make the following contributions in this paper:

- New understanding of the timing-based probing attack: We study common timing probing attacks and define a general behavior model to describe the timing probing attacks. Based on the proposed model, we generate behavior patterns corresponding to different timing probing attacks, respectively.
- Light-weight approach to expose and limit the timing-based probing attack: We propose an extension-based approach that monitors web application’s behaviors and detects timing probing attacks, based on the repeat rate of structured-probing-behavior patterns. Our approach makes it more difficult for the timing-base probing attacks to succeed.
- System prototype and evaluation: We prototyped our approach as an extension of Google Chrome. We also applied it on the Alexa Top 140,000 websites and 10,000 websites randomly selected from Alexa Top 1,000,000 websites and reported the suspicious behavior patterns with corresponding analysis results. We conducted theoretical analysis and demonstrated that the accuracy of the timing probing attacks is limited by our approach effectively.

## 2. Related Work

Attacks that allow attackers to directly affect the integrity of web applications, such as cross-site scripting [21] and cross-site request forgery [22], have been the main focus of web security research. A wide range of solutions has been developed to protect the security of web applications, such as [23,24,25,26,27,28]. Prokhorenko et al. [29] survey and classify existing protection techniques. These solutions prevent attackers from directly retrieving information across origins. As a result, attackers need to achieve their malicious goals through indirect attacks.

Side channel attacks in web applications, such as [30], have been shown to be an effectively way to passively infer information from web applications. As the attacker’s malicious JavaScript runs in the same browser environment as the victim web application, attackers can use more active techniques to infer information from web applications.

Kotcher et al. [14] discovered two timing probing attacks using CSS default filters. The first attack can check whether a user has an account with a website by exploiting the Document Object Model (DOM) rendering time difference. Additionally, the second attack can sniff user browsing history or read text tokens by stealing pixels on the user’s screen. They conducted evaluations of their attacks and proved the attacks’ feasibility.

Felten et al. [16] described a class of timing attacks used to sniff browsing history as well, but their attacks focus on operations whose time consumption depends on the cache status. They also proposed web cookies, which are a series of traditional cached files used as one traditional cookie. Using web cookies in attacks can make them more difficult to detect. To prevent these attacks, one can turn off caching or alter the hit or miss performance of the cache, but both will make caching less useful.

Weinberg et al. [15] proposed a way to sniff browsing history. The traditional way to do this is secretly checking the status of the browser environment. However, since browsing history will affect the appearance of the display to the user, the attacker can trick the user to tell him/her what the user has seen on the screen, which can be used to infer the user’s browsing history. The links are disguised as figures for users to recognize, so that while the user is recognizing the figures, he or she is telling the attacker his or her browsing history. They also come up with a way to “see” the user’s screen by monitoring the reflection of the screen using the user’s web camera. They proved that this is also a practical way to sniff the user’s browsing history.

Bansal et al. [31] exploited the Web Worker APIs to make the cache probing operation parallel, which speeds up the traditional cache probing attacks. They also proposed the idea of canceling resource requests once the attacker can confirm that the resource being probed is from browser cache. In this way, the attacker can avoid polluting the cache. They applied their improved cache timing attack on four scenarios, including attacks on web environment and operating systems. At the end of their paper, they discussed potential countermeasures, such as separating cache among different operating system components and setting no-cache headers to private data. However, the improved attacks do not reduce the number of probes that the attacker requires, so our approach can still detect these attacks.

Chen et al. [30] found a vulnerability in four web applications that can leak users’ sensitive information. The basis of this vulnerability is that, since the application needs to provide different contents according to the user’s choices, different user inputs will result in different network traffic sizes. Furthermore, usually the possible user inputs at one application state are very few, making it easy to guess the user’s input. The authors also use history traffic data to aid the process of guessing the user’s input. In their opinion, to mitigate effectively and efficiently the effect of this vulnerability, the method must be application-specific.

Following our early investigation [32], our approach aims to unify the characteristics of timing-based probing attacks. Based on the statistics and pattern of such attacks, we help to expose them to users and prevent them from extracting sensitive information in the background.

## 3. Overview

In this section, we discuss the threat from timing-based probing attacks and analyze their core features that can be used as the basis for detecting them.

### 3.1. Background

In this paper, we assume the adversary to be a web attacker. That is, the attacker controls a website and can run JavaScript in the victim’s browser. However, the attacker cannot run native code in the victim’s system, nor can the attacker exploit vulnerabilities in the victim’s system or browser. The attacker aims to infer the victim user’s private information in the browser environment through timing probing attacks. We aim to prevent user privacy leakage from shared environment states, which is different from data privacy attacks and defenses that focus on inference only from data [33,34].

#### 3.1.1. Timing-Based Probing Attacks

Felten and Schneider [16] introduce a timing attack to web applications. This attack measures the time required to load a web resource. As the time needed to load a web resource is affected by the resource’s cache status, the attacker can learn the resource’s cache status and then infer the user’s browsing history.

From this example, we generalize timing-based probing attacks as follows. The attacker first retrieves time from the system, which is either to record the system time or to start a timer. We refer to this activity as $T_{1}$ and the time obtained as the starting time $t_{1}$. It then starts a workload *W*, such as loading resources or performing a computation. Once *W* is finished, the attacker immediately takes another time measurement. We refer to this activity as $T_{2}$ and the time obtained as the ending time $t_{2}$. The time difference $t=t_{2}-t_{1}$ is the time spent on *W*. If *W* depends on browser states that cannot be directly accessed by attackers, *t* reveals information about such states.

#### 3.1.2. Properties of Timing Probing Attacks

Timing-based probing attacks are usually invisible to users [14,16,17,18]. Since they are launched to indirectly infer other users’ sensitive data with the presence of strong security mechanisms in browsers, each probing attempt typically infers only limited information. For example, in the above attack, every time, the attacker can only infer whether a web resource is cached, among tens of thousands of resources that may reveal users’ privacy. In other words, the “data rate” of leaked information in such probing attacks is very low. Owing to the limited information accessible through probing, attackers/malicious websites need a large number of repetitive operations to extract enough information for inferring a small number of users’ privacy. For example, to probe users’ browsing history, the attacker must prepare a list of URLs and check each of them repeatedly (using the behavior sequence $\left(T_{1},W,T_{2}\right)$). In addition, the result of a practical timing-based probing will differ depending on the speed of the hardware on which the browser is running. To achieve accurate time-based measurement results, attackers must repeat time measurement operations to achieve the desired calibration.

#### 3.1.3. Challenge

Though timing-based probing attacks do require repetitive behaviors of accessing time and carrying out the workload, benign web applications also have legitimate reasons to frequently access time and perform regular activities. Simply repeating such behaviors cannot be considered as the distinguished feature to identify the timing-based probing attacks. We need to distinguish benign repeated timing behaviors from malicious ones.

**Figure 1:**
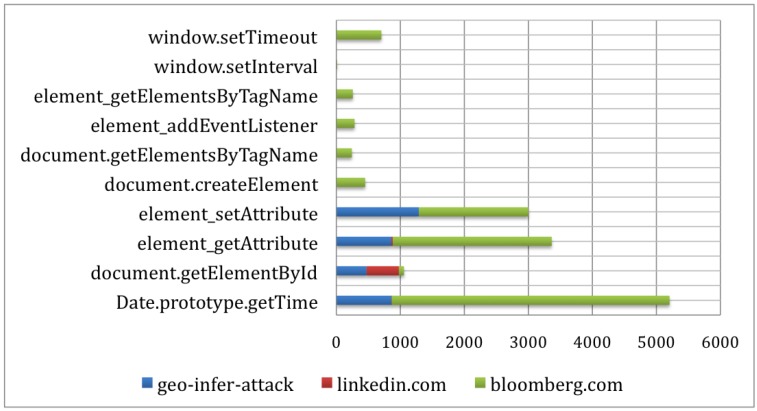
Distribution of the essential function calls.

To illustrate the problem, we monitored a set of important JavaScript APIs that are responsible for handling HTML elements and processing browser environment information. We measure the frequency the functions are called in web applications, which include websites with probing behaviors and Alexa Top 5000 websites. The selected experiment results shown in Figure 1 demonstrate that probing websites inevitably invoked some APIs to accumulate privacy information. For example, during the period of our measurement, Date.getTime() is invoked 860 times by a site preforming geo-location inference [18]; element_getAttribute() is called 860 times; and element_setAttribute() is called 1290 times. However, we also found benign websites calling such APIs in a relative high frequency. For example, *bloomberg.com* called Data.prototype.getTime() 4341 times, element_getAttribute() 2483 times, and element_setAttribute() 1709 times, respectively.

Therefore, the challenge is to distinguish the repetitive behavior of timing probing attacks, from that of legitimate web applications. From the generalized attack behavior described above, we can see that the distinguishing feature of timing-based probing attacks is the repetition of a group of behaviors $\left(T_{1},W,T_{2}\right)$, including a pair of actions to obtain the time and activities for the task. Detecting such behaviors is the intuition of our approach.

## 4. Design

**Figure 2:**
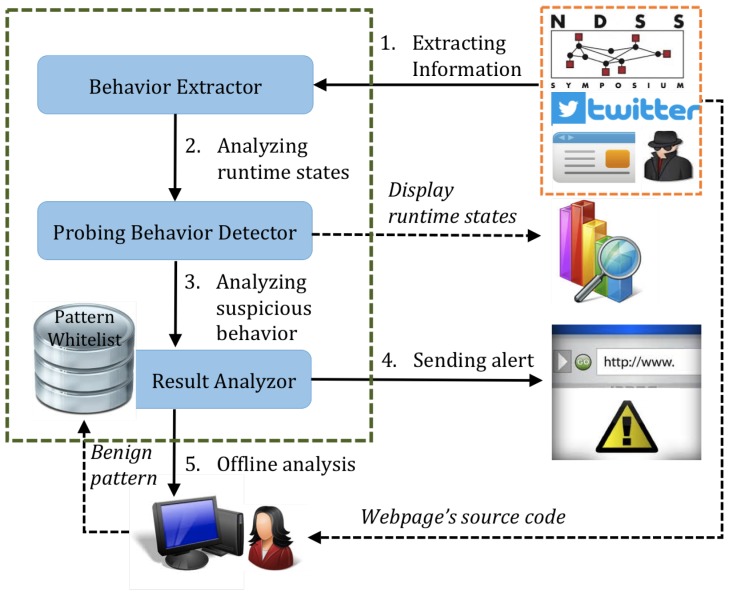
Architecture of our approach.

The overall architecture of our approach is illustrated in Figure 2. It monitors a web application’s behaviors for abnormal patterns of timing-related events to alert the user of potential timing-based probing attacks. Our approach consists of three key components: *Behavior Extractor*, *Probing Behavior Detector* and *Result Analyzer*.

In particular, the Behavior Extractor component monitors the web application and extracts its runtime execution information; the Probing Behavior Detector analyzes the runtime states gathered by the Behavior Extractor and detects suspicious timing-based probing behaviors; according to the knowledge in Pattern White List, Result Analyzer analyzes the suspicious behaviors detected by Probing Behavior Detector; once a malicious behavior is confirmed, it displays an alert to the browser user. The intercepted behaviors and detection results are made available to the Offline Analysis component, where analysts identifies benign repetitive timing behaviors and update the Pattern White List, so that such benign behaviors will not be flagged as attacks in the future.

### 4.1. Extracting Runtime Behaviors

Behavior extraction is the basis of our approach. Since most of a web application’s dynamic behaviors are carried out by JavaScript, our tool intercepts JavaScript API calls to represent the behavior of a web application. The behavior extractor module records the API together with its parameters. To help to understand the behavior and distinguish APIs used under different scenarios, the behavior extractor also extracts the runtime JavaScript stack of the API.

To extract these runtime behaviors without modifying the browser, we build our tool as a browser extension. Though our focus is mainly on the timing-related behaviors, the interception mechanism is flexible to allow users to specify general JavaScript APIs to monitor. It intercepts JavaScript behaviors of web applications through rewriting JavaScript functions. Our extension preloads the rewriting JavaScript code before any code in the web application is loaded. It then takes a list of APIs to be hooked, which is specified by the users, and interposes the APIs to output them in a log of JavaScript behaviors, which record the functions’ names, the arguments and the functions’ call stack information.

### 4.2. Detecting Probing Behaviors

Timing-related APIs are commonly used in benign web applications. To distinguish benign usage of timing APIs from probing attacks, the probing behavior detector adopts a two-level detection mechanism to expose probing behaviors. First, it flags suspicious timing behaviors based on the frequency pattern, i.e., the frequency of timing behaviors and their distribution over time. It serves as the first-level detector. To further prevent mistakenly reporting benign timing behaviors as probing attacks, the detector analyzes the structure of timing behaviors and performs detection based on structured timing behavior pattern.

#### 4.2.1. Distribution-Based Probing Behavior Pattern

The first-level detection is based on the distribution of timing-behaviors. We gather statistics on how many times timing functions are called over a period of time.

At this level, our approach provides light-weight real-time attack detection. Taking the cache timing attack explained above as an example, our approach displays statistics for monitored JavaScript APIs. The user can pay attention to the APIs that obtain times from the system. Once the frequency exceeds a certain threshold, the user can be alarmed about possible timing attack on the website he is visiting. To further increase the detection accuracy, we use another level detection via structure information of web application’s timing behavior.

#### 4.2.2. Structure-Based Probing Behavior Pattern

Timing probing attacks always contain two operations of obtaining system time value $T_{1}$ and $T_{2}$, as well as a workload in the middle *W*, which is the operation to be timed. Therefore, it forms the following pattern:

1. A get-time activity $T_{1}$ (such as a Date.getTime() API call or an event to set a timer);
2. One or several workload operations, such as loading an image or drawing a frame. We refer to the collection of such operations as *W*; Note that the behaviors in *W* might not always be shown as the form of a function call (e.g., when loading an image, it is just an assignment to the src attribute of the image element);
3. Another get-time operation $T_{2}$.

Due to the asynchronous nature of the JavaScript language, $T_{1}$ and $T_{2}$ often appear in completely different program locations and contexts. However, in many timing probing attacks, *W* is carried out by an asynchronous operation, where $T_{2}$ can only be obtained in the callback functions of the asynchronous operation. When $T_{2}$ is executed, it is difficult to decide the corresponding $T_{1}$ and *W*. Moreover, in actual timing attacks, the $\left(T_{1},W,T_{2}\right)$ pattern may repeat multiple times consecutively, effective making $T_{2}$ of one iteration the first get-time event of later iterations, resulting in the following pattern $\left(T_{1},W,T_{2},W^{\prime },T_{2}^{\prime },\ldots \right)$. In this case, we can use the connection between the last two operations as our pattern for the timing attack and treat the abnormal amount of the repeated pattern as an indicator of a timing attack.

Some attacks that have distinguishing features that can be identified inside the behavior log can be detected automatically. Take the cache timing attack as an example. This attack always contains two system time acquire operations (normally by calling the Date.getTime function), one before a resource (often an image) begins to load and another after the resource is loaded (i.e., called inside the onload function of the resource). For this attack, the connection between $T_{2}$ and *W* is the Date.getTime function call for $T_{2}$ is inside an onload function of an HTMLImageELement for *W*. This connection, between the second system time acquire operation $T_{2}$ and the image loading operation *W*, plays an important role in distinguishing benign behaviors from probing behaviors in websites in our experiment.

## 5. Implementation

**Figure 3:**
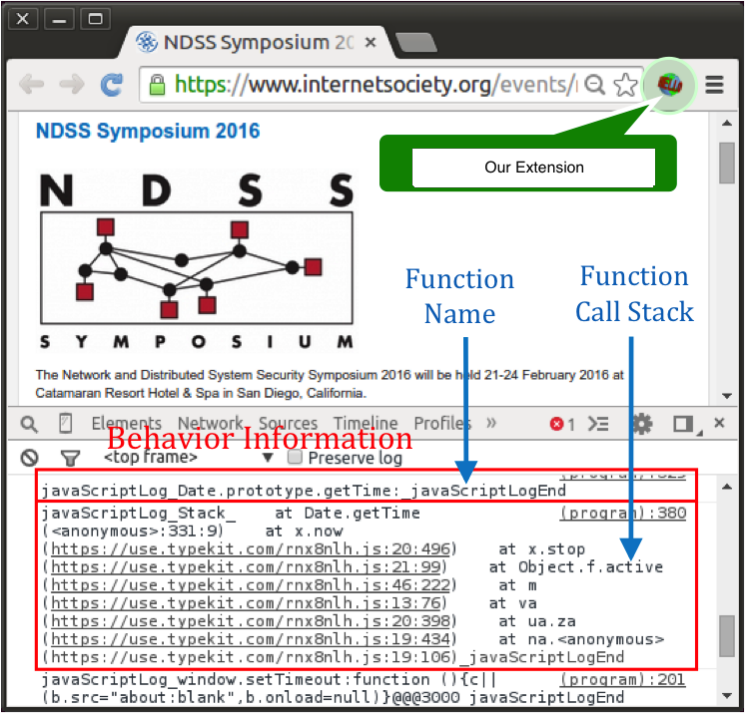
Runtime snapshot of our approach. Website behavior information is logged into the browser console.

We have prototyped our approach as an extension to the Google Chrome browser. Our prototype is based on the 64-bit Chrome browser (Version 42.0.2311.90). Figure 3 shows a runtime snapshot of the implementation. In Figure 3, our extension is displayed at the right side of the address bar. The website behavior information is logged into browser console, which includes critical function names and function call stacks. In this section, we describe the challenges we addressed in the implementation and introduce our system in detail.

### 5.1. Behavior Extraction

Our approach intercepts JavaScript behaviors of web applications through rewriting JavaScript functions. Our extension preloads the rewriting JavaScript code before any code in the web application is loaded. It then takes a list of APIs to be hooked, which is specified by the users, and interposes the APIs to output them in a log of JavaScript behaviors.

To intercept the information about function calls, our method is to interpose a set of new functions on top of the APIs to be intercepted. First, we need to make sure all calls to the intercepted APIs are handled by the new functions first, instead of the original ones. Our solution is to rename the original API and use the new functions as the API.

The newly-defined functions will invoke the original API inside the newly-inserted functions. The difficulty in this part is to deliver all arguments completely and correctly, to make the interposition transparent to web applications. JavaScript functions with no arguments declared can take any amount of arguments, and the taken arguments can be accessed inside the function using the variable arguments. Besides, all JavaScript functions have a member function named apply, which can take arguments in the form of variable arguments. Therefore, using these two features, we can pass the arguments transparently.

Now that we have interposed API functions, we can extract the necessary information inside the inserted functions. Following the design of the behavior extraction, we record the function names, the arguments and the functions’ call stack information. The function-name extraction can be easily done by adding function names into the inserted functions. Furthermore, the function arguments can be obtained inside the inserted functions naturally.

While obtaining API call information is straightforward, getting JavaScript call stack information needs more effort. We exploit an error-handling feature of JavaScript, by triggering a JavaScript error to get a function’s call stack information. Additionally, if the error happens inside a “try” block and taken good care of inside the corresponding “catch” block, the web application will not be affected.

### 5.2. Probing Behavior Detection

Our behavior extension provides real-time behavior information. It helps to expose probing behaviors to knowledgeable users.

One kind of information we provide is the times of JavaScript system functions are called. To count the times all JavaScript system functions are called, we add counters for each function. On one hand, these counters must be able to be accessed by the host web page, to be modified when the corresponding function is called. On the other hand, these counters must be able to be accessed by the extension (more specifically, the content scripts of the extension) as well, in order to be used in drawing charts. There is only one thing that can be accessed both by the host web page and the extension, which is HTML elements. Therefore, we create a list of HTML elements, add an attribute named count with initial value zero to each of the HTML elements and set their id values to be the functions’ names. When a function is called, we will get the corresponding HTML element using the id attribute and add one to the value of the count attribute to record this function call. In this way, we use a list of HTML elements as counters to count the times the functions are called. We also append a 16-digit random number to each id values, just to avoid conflict.

We use a similar method for the element access count. Note that we call an element accessed when the element’s member function is called. Therefore, we only need to change our focus from JavaScript system functions to HTML elements’ member functions. Once an HTML element’s member function is called, we get the corresponding counter (the value of an attribute of an HTML element) and add one to it, to represent one access of this HTML element. We also use the same method to avoid conflict in id values.

**Figure 4:**
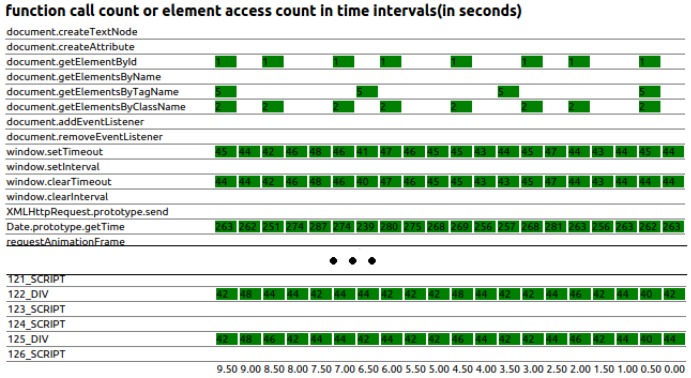
This chart illustrates the real-time states, including the number of function calls and HTML element accesses in time intervals.

To avoid disturbing the user, we cannot directly draw the chart on top of the web page. Instead, we draw the chart in the pocket-size web page managed by the extension. Before drawing the chart, we need to send the counters values from content_scripts to this pocket-size web page, which can be done by using the extension’s built-in communication method onRequest and onMessage. Additionally, for the drawing part, we choose to use a simple, but practical way, which is to create a <table> element and modify the elements’ value and their background colors. We get counter values as a group every few seconds and calculate the difference between values of two adjacent groups. Then, we write the calculated results to the elements of the right-most column of the table. We also set background colors of elements whose value is not zero, to address newly-appearing function calls or element accesses. In this way, our charts show function calls and element accesses in the past few seconds, as well as the history data used to compare with the latest ones. Figure 4 visually illustrates the real-time states of the testing website, which includes the number of functions calls and HTML element accesses in specific intervals (in this example, we counted in 0.5 s). The difference of the counter values in a time interval is shown in the green block, and the corresponding time interval is listed on the bottom of the chart. The blank part represents that the counter value difference is zero in the current time interval.

**Figure 5:**
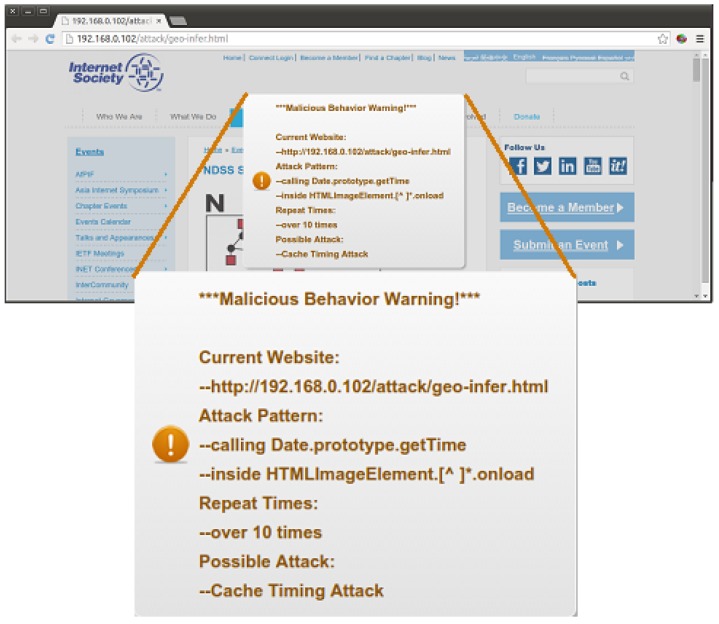
Given a malicious website, once the probing behavior detector finds suspicious behaviors, it raises an alert to warn the user.

Given a web application, we calculate the times of successful pattern matching at runtime and a pop-up warning message once the counter value exceeds a certain threshold. Figure 5 displays how it works when the detector finds suspicious behaviors. The warning information shown in the system alert includes the URL of the suspicious website, attack pattern matched, repeat times, possible attack, etc.

### 5.3. Result Analysis and Offline Analyzer

We build a pattern white-list database, which stores information of confirmed benign websites. The information used as white-list data can be the URL of the website, the name of the script conducting the suspicious function calls or even the function call stack when suspicious functions are called. These types of information will result in different effects on the white-list mechanism, which is suitable for different situations. We use this white-list data to reduce the false alarm rate in probing behavior detection.

We also use the console.log function to record the behavior information to the browser console, to allow the analyzer to conduct offline behavior analysis. To distinguish our behavior logs from the websites’ own console logs, we encircle our behavior logs with marking strings. Since the offline behavior analysis requires the behavior logs written in a file, we use the –enable-logging option of the Chrome browser to write the console logs to a file named chrome_debug.log.

To confirm the correctness of the attack warnings, we manually check the behavior log files, even the source code of all of the websites with many pattern matchings. Once we rule out the possibility of timing probing attacks, we update this website’s information into the white-list database, to avoid reporting this website as malicious in the future.

### 5.4. Automatic Parallel Testing Method

To speed up the website testing on a large number of websites, we present an automatic parallel testing method.

To move reliably on to the next test subject after testing the current test subject, we designed a three-fold auto shutdown mechanism, which will shutdown the browser when any of these situations happen: (a) the website body element’s onload event is triggered; (b) the website has been opened for a certain time; (c) the browser has run for a certain time. Note that we only open one website (i.e., one browsing tab) in one browser window and have already set the browser to close itself when all tabs are closed, so when the website inside the browser closes, the browser will be closed as well.

The design of the single-processing mode testing is relatively straight-forward: simply reading a website URL from a URL list file, opening this website and repeating the above two steps, until all URLs in the list are tested. It is noteworthy that a check is made of the log’s content after the browser is shut down, and the useless ones are discarded to ensure all behavior logs left are meaningful.

To make full use of the hardware computing power to reduce time consumption, a multiple-process mode to test websites in a parallel manner was designed. The first process this mode needs to accomplish is to prepare workspaces for each single-process mode test. The Chrome browser uses user-date-dir to remember user settings like installed extensions, and to identify independent browser instances. Consequently, we make copies of the user-date-dir directory for all single-process mode tests. In this way, each single-process mode test will open a browser with our behavior extension already installed, in the mean time independent from other browser windows, so that two websites’ behavior logs will not be mixed.

## 6. Evaluation

We have evaluated our approach from the following aspects. We first used recent timing probing attacks and showed that our approach can successfully detect them. We then used our approach to scan the top 140,000 sites from Alexa, randomly selected 10,000 websites from the Alexa Top 1,000,000 websites and identify sites with potential probing behavior. We also used an empirical measurement to demonstrate how our approach limits the rate of successful attacks. Finally, we show the performance overhead of our approach.

### 6.1. Existing Timing Probing Attacks Detection

To evaluate the effectiveness of our approach, we used recent timing probing attacks in web applications and implemented the attacks according to their technical descriptions. We first introduce the attacks and then describe how they are exposed by our approach.

#### 6.1.1. Web Caching-Based Timing Probing Attack

**Figure 6:**
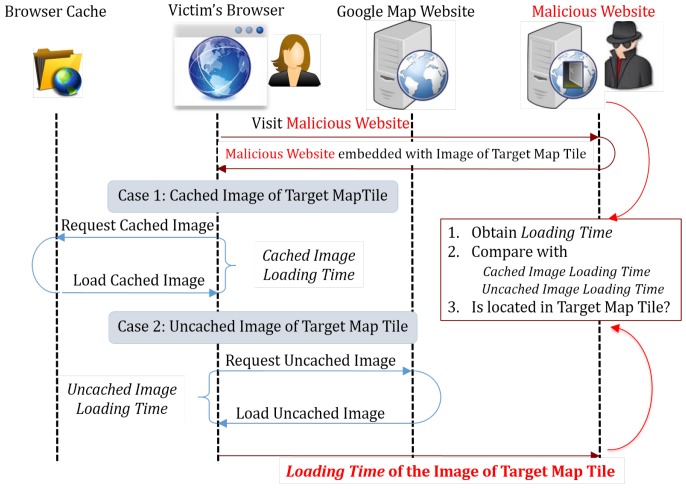
Web caching-based timing probing attack.

The web browser uses caching to speed up the access to the recently visited files/resources. A web-caching-based probing attack may make use of this functionality by measuring the time required to access a specific file belonging to another origin. If that file is in the user’s cache, the access must be fast. Otherwise, the access will be slow. According to the time cost for accessing, the attacker may infer whether the file is in the browser’s cache and whether the user has accessed the target origin as well (namely, being able to deduce the users’ browsing history indirectly). We illustrate the basic principle of the cache-based probing attack in Figure 6. To evaluate the effectiveness of the web caching-based attack, we use the attack proposed by Jia et al. [18] as the test case.

In this attack, a malicious web page needs to measure the loading time of image files, so it is required to call the Date.getTime function after the image is loaded. The corresponding behavior pattern is calling the Date.getTime function inside the onload function of the HTMLImageElement element. The pattern matching can be done by searching for lines containing both string Date.getTime and regular expression “HTMLImageElement∖.[^ ]*∖.onload” inside the function call stack data.

**Figure 7:**
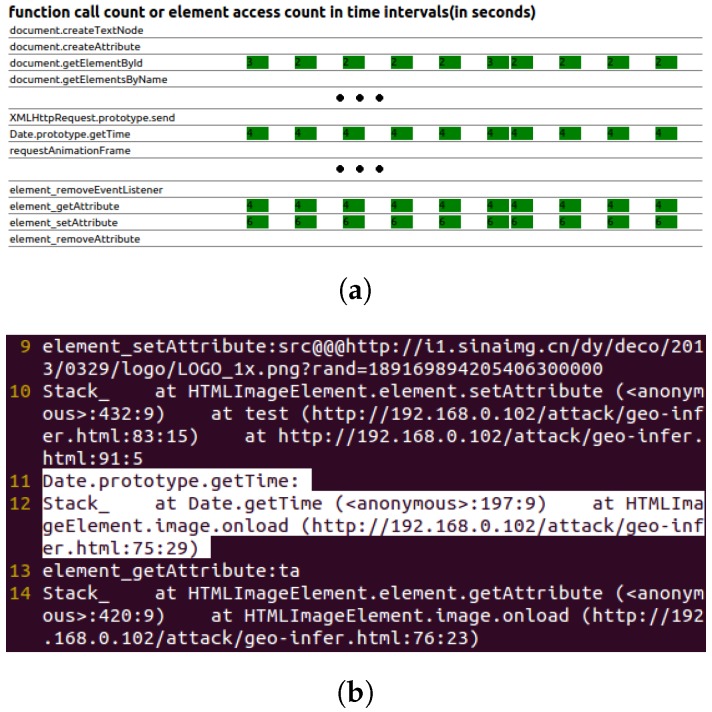
Web caching-based timing probing attack. (**a**) Real-time function call and element access chart; (**b**) behavior log (attack pattern is highlighted).

Figure 7 illustrates the chart for real-time function call and element access (first-level detection), as well as the key behavior logs of the web-caching-based timing probing attack (second-level detection).

#### 6.1.2. Pixel-Based Timing Probing Attack

Kotcher et al. [14] proposed another timing probing attack, which allows the attacker to “see” the target website.

The shader inside browsers often takes different times to draw pixels of different colors. In theory, by measuring the time took to draw a pixel, the attacker can know the color of this pixel. Additionally, by traversing all pixels inside a target region of the target website, the attacker can get an image of this region, i.e., “see” this region of the target website.

Then, the attacker will measure the web page drawing time to infer the color of the pixels, and this can be done by measuring the refreshing rate of the web page. By consecutively calling the requestAnimationFrame function until a certain amount of frames have been drawn and measuring the time required to draw these frames, the attacker can know the web page’s refreshing rate, which is mostly influenced by the drawing speed of pixels of different colors. Therefore, by checking the web page’s refreshing rate, the attacker can know the color of the current targeting pixel.

**Figure 8:**
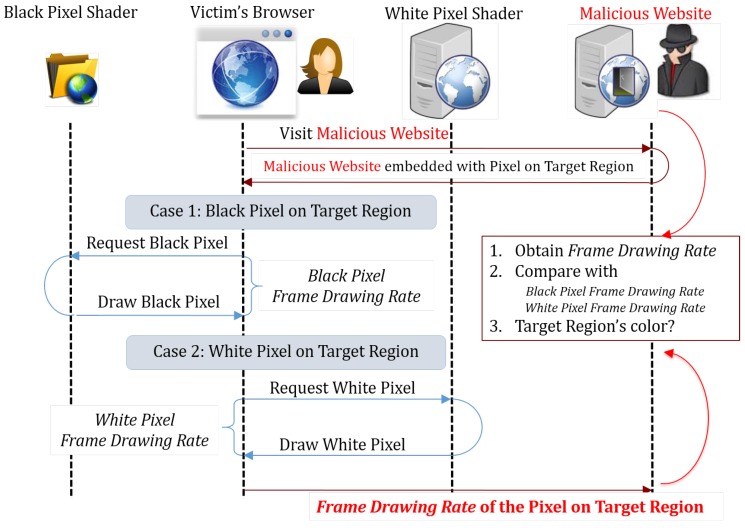
Pixel-based timing probing attack.

Figure 8 shows how the attack works. The attacker first prepares a list of coordinates of pixels to be tested and makes the pixels inside the target region either black or white. Then, he/she selects pixels one by one, enlarges them to full-screen size and measures the web page’s refreshing rate. After all of the pixels are tested, the refreshing rate data of all pixels are sent back to the attacker’s web server, so that the attacker can infer the color of each pixel and draw the image of the target region.

**Figure 9:**
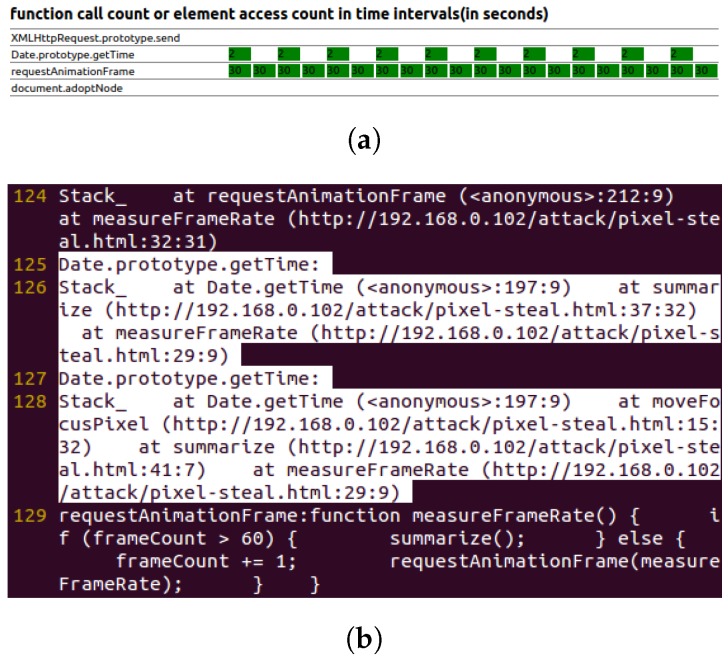
Pixel-based timing probing attack. (**a**) Real-time function call and element access chart; (**b**) behavior log (attack pattern is highlighted).

Figure 9 shows the real-time function call and element access chart, as well as the key behavior log of the pixel-based timing probing attack.

The attack needs to measure the frame rate of websites, which needs to call the Date.getTime function after a frame is drawn. Additionally, the function used as the requestAnimationFrame function arguments will be called after a frame is drawn. The pattern used to indicate this attack is calling the Date.getTime function inside functions that are used as the requestAnimationFrame function’s argument. The pattern matching can be done by first searching for regular expression “^ requestAnimationFrame: function [^ (]*(” inside function call data and getting the argument function’s names, then searching for lines containing both string Date.getTime and the argument function’s names, inside the function call stack data.

#### 6.1.3. Repainting-Based Timing Probing Attack

Stone [17] proposed a way to check links’ visit status and sniff the browsing history by measuring the web page’s repainting time.

According to the synchronizing property of the Chrome browser, when the browser found that a link’s target URL has changed, it will check whether this link’s visit status has changed as well, e.g., from unvisited to visited. If the visit status has changed, the Chrome browser will repaint this link element, while doing nothing if the visit status remains the same. The repainting operation may be inferred by measuring the drawing time of the web page.

**Figure 10:**
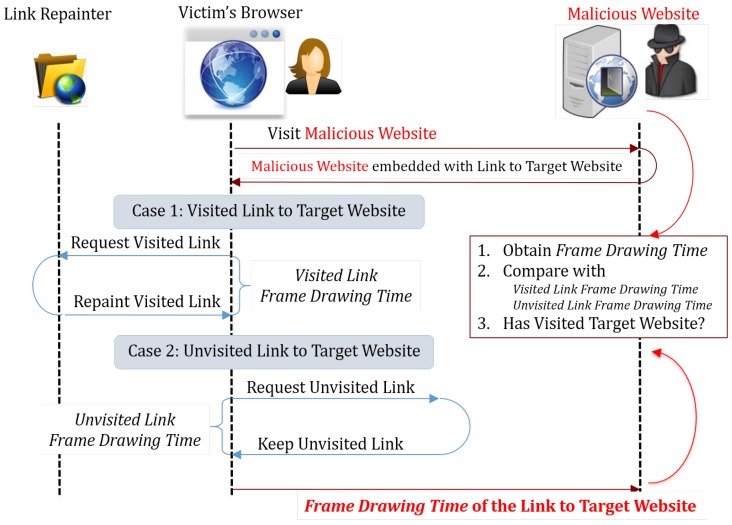
Repainting-based timing probing attack.

Figure 10 illustrates the URL-repainting time-based history sniffing attack. To know whether a certain website has been visited by the victim, the attacker will first make a link pointing to a fake URL that is guaranteed to be unvisited. Then, the attacker changes this link and lets it point to the target website URL. The attacker may deduce whether the link’s visit status has changed by measuring the time required to draw the next frame and know whether the target website has been visited by the victim or not.

**Figure 11:**
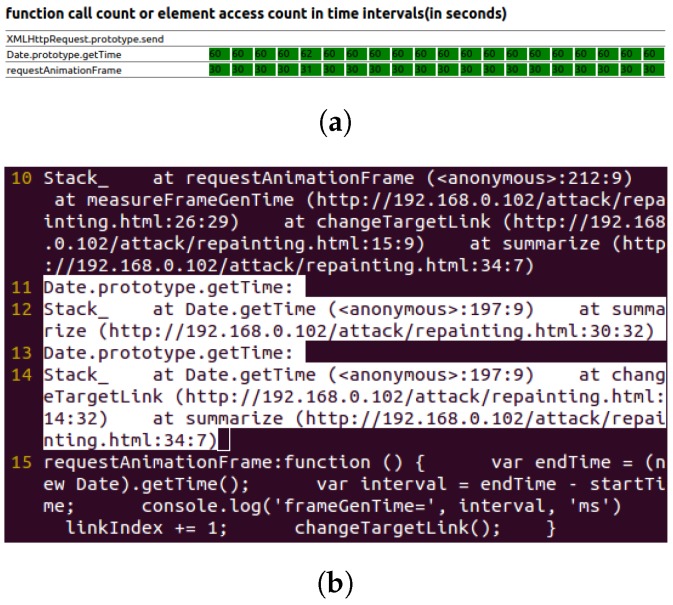
Repainting based timing probing attack. (**a**) Real-time function call and element access chart; (**b**) behavior log (attack pattern is highlighted).

Figure 11 shows the real-time function call and element access chart, as well as the key behavior log of the repainting-based timing probing attack.

This attack needs to measure the frame drawing time of a certain frame, which needs to call the Date.getTime function after a frame is drawn, the same as the last attack. Note that the function used as requestAnimationFrame function’s argument will be called after a frame is drawn. Thus, the pattern used to indicate this attack is still calling the Date.getTime function inside functions that are used as the requestAnimationFrame function’s argument. The pattern matching can be done the same way as in the last attack. Additionally, the pattern distribution is the same as in the last attack as well.

### 6.2. Web Applications’ Behavior Analysis

**Figure 12:**
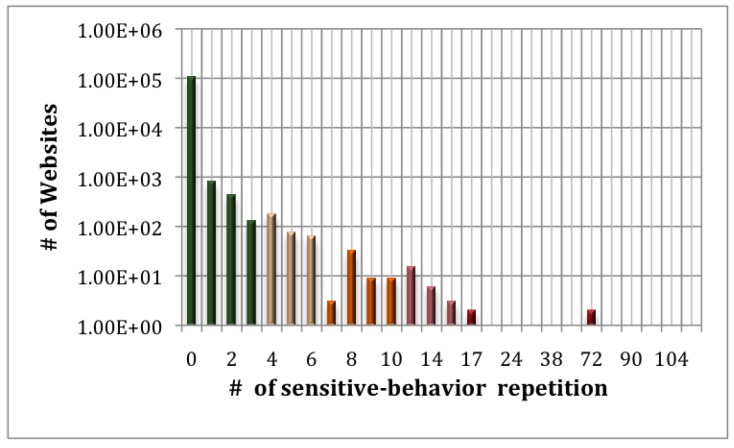
Amount of websites that match the web cache-based timing probing attack pattern for a certain time.

We select the Alexa [35] Top 140,000 websites (106,985 valid) and test their cache-timing based probing behaviors. As shown in Figure 12, only 47 websites repeat the cache-timing based probing pattern more than 10 times.

**Figure 13:**
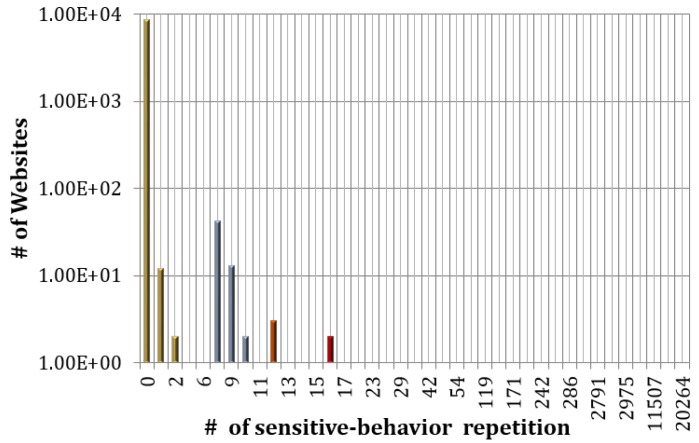
Amount of websites that match pixel-based timing probing attack patterns for a certain time.

We also conduct the experiment on 10,000 randomly selected websites among the Alexa Top 1,000,000 websites (8695 valid results) to test the pixel-based probing behavior and summarize the result in Figure 13. As shown in Figure 13, only 36 websites have the pixel-based probing pattern over 10 times. According to the testing result, we suggest to use the experiential value, 10, as the detecting threshold, and the experiments also demonstrate that our approach will not affect most of the normal websites.

We manually checked run-time behavior logs and the source code of the websites that have certain suspicious behavior patterns over the detecting threshold. The offline analysis result shows that the suspicious behavior patterns in these websites are benign/reasonable operations. We extract the auxiliary information to white-list the benign behavior. The benign behaviors may be divided into the following categories.

1. Checking network connection status: These websites measure the images’ loading time and send it back to a remote server. They append a random number at the end of an image’s URL, e.g.: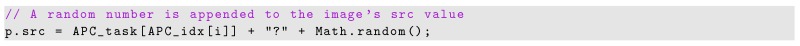  so that these images will not be loaded from browser cache. Moreover, the images are from the same origin as the websites, and the testing result is also sent back to their servers. For example, the part of the source code of *qq.com* is:
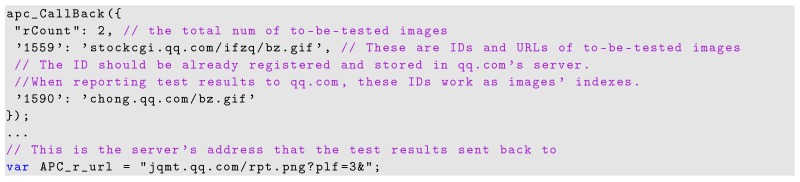
  This makes it a cache-based timing probing attack.
  In this case, the websites cannot infer the cached status of the images, which means the repeated operations are not targeting to probe user’s privacy information.
2. Setting cookies: Some websites usually conduct some irrelevant tests first and return the results to the server via arguments of the URL of an image. To avoid conducting the same test within a period, they get the system time after the results are sent and set the cookie’s expire time based on this value. For example,
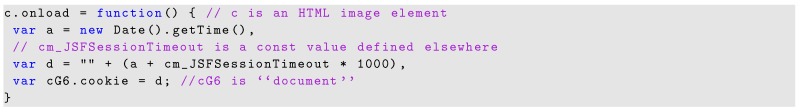
3. Discarding obtained time: Some websites include the jQuery script. Inside this script, the system time value is obtained by calling the Date.getTime function. However, the obtained time value is never used in the following process, i.e.,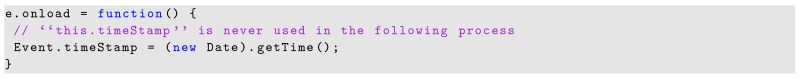  We treat this as a default operation by jQuery script or it may be for future updates.
4. Drawing animation: These websites obtain system time in order to update animations after a certain time. Before the time is up, it allows the browser to draw several frames. Additionally, after this certain time, the website changes HTML elements’ CSS attributes, and restarts the above process. This is an example piece of code from websites in this category:
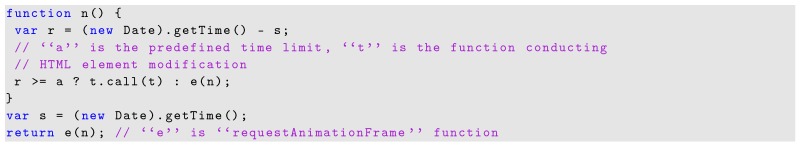
5. Progress Indicator: The websites in this category obtain system time value to calculate the process finishing status. The system time is compared with predefined starting and finishing time, and by calculating the position of the current time between these two time values, the program can get the progress status. An example piece of the code is shown below:
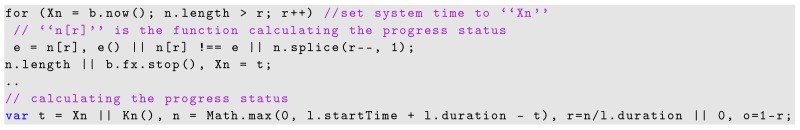

We summarize the white-listed behaviors based on the function call stack information of the benign websites. Table 1 and Table 2 illustrate the benign behavior categories with the behavior representation and corresponding websites white-listed.

### 6.3. Rate-Limiting Probing Attacks

According to the design of our approach, attackers have to limit their probing frequencies to avoid being detected. In this subsection, we theoretically analyze the rate-limiting effect on existing probing attacks.

#### 6.3.1. Web Caching-Based Timing Probing Attack

Considering the attack discussed in Jia’s work [18]; each time the attacker probes, i.e., measures one image’s loading time, the attacker can either locate the victim in a certain map tile or not do so. Suppose there are *m* map tiles that the attacker can locate the victim in by conducting the probing once, and the attacker can only probe *n* times before being caught by our approach. We also suppose the probability of the victim located in different map tiles is the same, so that the attacker has to randomly select *n* map tiles to conduct probes. The probability of the victim being located in one certain map tile can be calculated as $p=\frac{1}{m}$.

We first calculate the probability of successfully locating the victim when the victim’s location is fixed, which is equivalent to having probed the image of the map tile in which the victim is located within *n* probes. The probability can be calculated as:

$$P_{fix}=\frac{C_{1}^{1}\cdot C_{m-1}^{n-1}}{C_{m}^{n}}=\frac{n}{m}.$$

Since the victim’s location can belong to any map tiles, we calculate the weighted average value of $p_{fix}$, where the weight value is the probability of the victim located in one certain map tile, which is $p=\frac{1}{m}$. Therefore, the average probability of a successful attack can be calculated as:

$$P=\sum_{i=1}^{m}P_{fix}\cdot p=\frac{n}{m}.$$

Note that in this attack, the attacker needs at least four times of probing for calibration [16] before starting the cache-status probing, which will also be counted by our approach. Therefore, considering this calibration step, the average probability of a successful attack, *P*, should be $P=\frac{n-4}{m}$.

**Figure 14:**
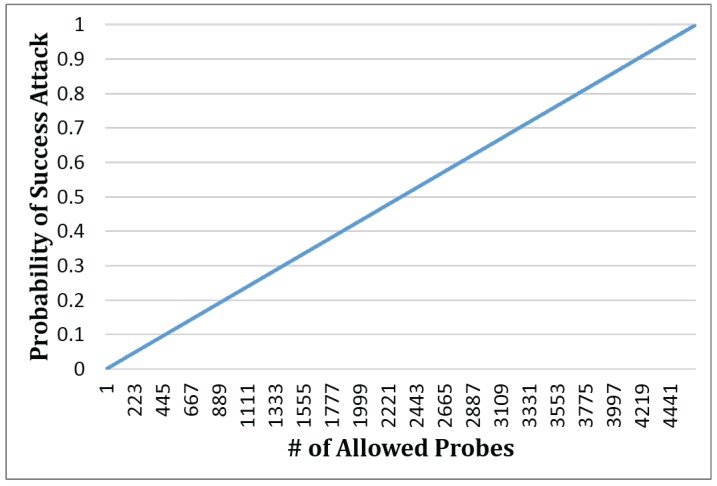
Probability of successful attack with different amounts of probes.

In the practical attack discussed by Jia et al. [18], the amount of map tiles of New York City is 4646, that is m=4646. The probability of a successful attack with *n* times probing is shown in Figure 14. According to the Figure 14, if the detecting threshold is set as 10, the success rate will drop from 100% to 0.13%, compared to unlimited probing times.

#### 6.3.2. Pixel-Based Timing Probing Attack

For pixel-based timing probing attack, to recover the original image with a limited amount of pixels, the best way is to uniformly spread the to-be-probed pixels on the original image. We suppose that the attacker will choose pixels to probe uniformly distributed in a square, and the width-height-ratio is similar to the original image. Additionally, to recovery the original image with probed pixels, interpolation [36] is the best choice. We choose the bicubic interpolation process [37] to recover the sampled image, because it has the best recover effect among common interpolation processes (such as nearest-neighbor interpolation [38] or bilinear interpolation [39]) on our example image. Finally, we use peak signal to noise ratio (PSNR) [40] to evaluate the recover effect, which is a common metric to evaluate the quality of reconstructing lossy image. The PSNR is often defined based on mean squared error (MSE) calculated by the following formula.

$$MSR=\frac{1}{mn}\sum_{i=0}^{m-1}\sum_{j=0}^{n-1}{\left[I\left(i,j\right)-K\left(i,j\right)\right]}^{2},$$

where *I* and *K* are the original image and recovered image, respectively, both m×n pixels. Additionally, I(i,j) and K(i,j) represent the brightness value of the pixel in row *i* and column *j*. Therefore, the PSNR is defined as:

$$PSNR=20log_{10}\left(\frac{MAX_{I}}{\sqrt{MSE}}\right),$$

where $MAX_{I}$ is the maximum possible pixel value of the image, which equals two in black-white-only images.

**Figure 15:**
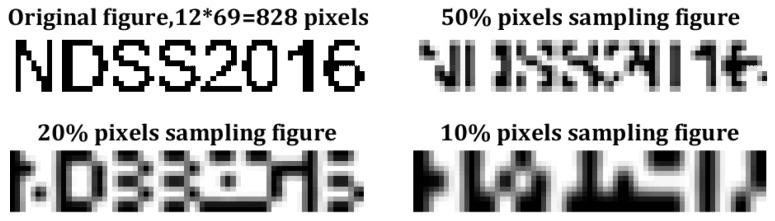
Image recovery result based on different amounts of pixels.

**Figure 16:**
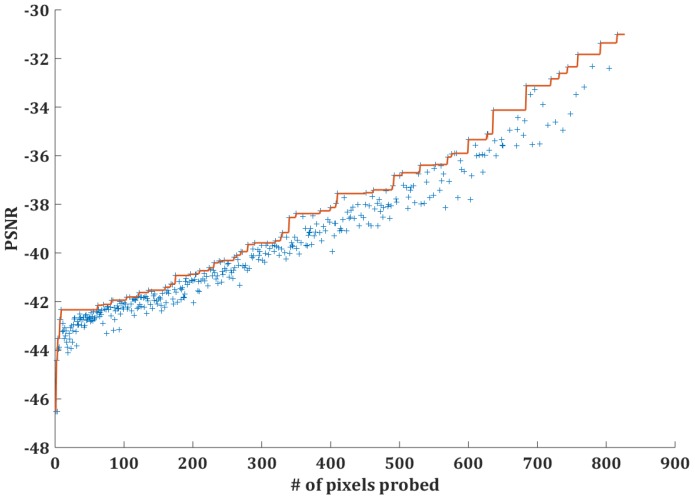
PSNR corresponding to different amounts of pixels.

We select a sample image, shown in Figure 15, as the original image the attacker wants to probe, sample it in every possible sample point distribution; use the interpolation method to recover the original image; and use PSNR value as the metric of the recovery effect. Figure 15 shows the example image and the recovered image based on different numbers of uniformly-distributed pixels. The blue crosses in Figure 16 show the PSNR value of the recovery based on different numbers of pixels, and the red step curve shows that the best PSNR value can be achieved based on different numbers of pixels. From this figure, we can see that limiting the number of pixels that the attacker can probe will influence the image recovery effect significantly.

#### 6.3.3. Repainting-Based Timing Probing Attacks

The timing of the repainting will be different relying on the hardware speed where the browser is running. To probe the browsing history accurately in different browser environments, calibration should be the first step before a real repainting-based timing probing attacks. The target of calibration is to determine the suitable values that make the painting slow enough to time with the requestAnimationFrame function and fast enough to check a large amount of link. Twenty probing executions are suggested in [17] to initiate a calibration phase. This is already above the common threshold used in our approach. Therefore, repainting-based timing probing attacks cannot conduct the calibration without being detected. As a result, the accuracy will be influenced significantly.

### 6.4. Performance

We evaluate the performance overhead of our approach. We test the average time cost of *baidu.com* under different configurations. The result is shown in Table 3.

The first three configurations are for offline behavior analysis. The additional overhead only affects the speed of evaluating a large number of websites, which can be optimized by our the automatic website parallel testing method. The last three configurations are for real-time behavior analysis. Drawing the real-time behavior chart causes negligible overhead. Additionally, with warning functionality enabled, the time cost only increases to approximately 1 s. This is only the starting phase of visiting a website; after the website has been loaded and stabilized, the difference can be neglected as well; users will not feel any difference.

## 7. Conclusions

While security mechanisms on the web platform prevent attackers from directly obtaining information to which they should not have access, attackers have turned to indirect attacks, inferring information from shared states in the browser. In this paper, we studied common browser probing attacks over the timing channel. We observe that time attacks typically need a large amount of operations to infer useful information. Based on this observation, we develop a solution to detect such attacks based on generalized behavior patterns. We present a browser-extension-based tool to detect browser probing attacks implemented in the Google Chrome browser. Our approach enables users to be aware of the potential risk of privacy leakage during their surfing activities and exposes the suspicious behaviors embedded in a malicious web page.

## Figures and Tables

**Table 1:** white-listed behavior patterns of web caching-based timing probing attack.

Category	Benign Pattern	Example Websites (Alexa No.)
Checking	Date.getTime	qq.com(10)
Connection	*at* APC_r_ok;	longzhu.tv(12874)
Status	*at* HTMLImageElement.APC_st.p.onload;	5068.com(28444) ⋯
	Date.getTime	
Setting	*at* cmJSFSetSingleSessionCookie;	teknosa.com(5805)
Cookies	*at* cmJSFSetSessionCookies; *at* C0; *at* c6;	ca-norddefrance.fr(20454)
	*at* HTMLImageElement.CV.c.onload;	frontgate.com(28130) ⋯
	Date.getTime	
	*at* Function.p.extend.now; *at* Object.p.Event;	
Discarding	*at* Object.p.event.trigger; *at* e;	kizi.com(1537)
Obtained	*at* p.fn.extend.triggerHandler;	keepcalm-o-matic.co.uk(19629)
Time	*at* Function.e.cleanData; *at* p.fn.extend.remove;	boxtv.com(38905) ⋯
	*at* HTMLImageElement.scrollHp.lastRenderedIndex.	
	game_hash.img_obj.onload;	

**Table 2:** white-listed behavior patterns of pixel- and repainting-based timing probing attack.

Category	Benign Pattern	Example Websites (Alexa No.)
Drawing animation	Date.getTime *at* Function.b.extend.now *at* Function.b.fx.tick *at* a	thedieline.com(1653) reinia.net(2301) ⋯
Progress Indicator	Date.getTime *at* Object.o.requestTimeout *at* Function.f *at* n	brahminsaptapadi.com(1453) vpncreative(3909) ⋯

**Table 3:** Time cost of different configurations.

Performance Test Environment	
**CPU:** Intel®Core™i5-4570	
**Memory:** 4 Gigabyte	
**Operating system:** Linux 12.04.2 (64-bit)	
**Browser:** Chrome, Version 42.0.2311.90 (64-bit)	
**Enabled Functionalities**	**Time Cost**
None	241 ms
Write function name to console	454 ms
Write function call stack to console	1420 ms
Write function name to console Write function call stack to console	1583 ms
Draw real-time behavior chart	310 ms
Warn attack (white-list enabled)	1180 ms
Draw real-time behavior chart Warn attack (white-list enabled)	1193 ms

## References

[1] Echevarria J.J., Ruiz-de Gariby J., Legarda J., Álvarez M., Ayerbe A., Vazquez J.I. (2012). WebTag: Web browsing into sensor tags over NFC. Sensors.

[2] Ji Z., Ganchev I., O’Droma M., Zhao L., Zhang X. (2014). A Cloud-Based Car Parking Middleware for IoT-based Smart Cities: Design and Implementation. Sensors.

[3] Miranda J., Cabral J., Wagner S., Fischer Pedersen C., Ravelo B., Memon M., Mathiesen M. (2016). An Open Platform for Seamless Sensor Support in Healthcare for the Internet of Things. Sensors.

[4] DOrazio C.J., Choo K.K.R., Yang L.Y. (2016). Data Exfiltration from Internet of Things Devices: iOS Devices as Case Studies. IEEE Internet Things J..

[5] Same-Origin Policy. https://developer.mozilla.org/en-US/docs/Web/Security/Same-origin_policy.

[6] Janc A., Olejnik L. Feasibility and Real-World Implications of Web Browser History Detection. Proceedings of the Web 2.0 Security and Privacy Workshop.

[7] Lee S., Kim H., Kim J. Identifying Cross-origin Resource Status Using Application Cache. Proceedings of the 2015 Network and Distributed System Security Symposium.

[8] Cabuk S., Brodley C.E., Shields C. IP Covert Timing Channels: Design and Detection. Proceedings of the 11th ACM Conference on Computer and Communications Security.

[9] Chevallier-Mames B., Ciet M., Joye M. (2004). Low-cost solutions for preventing simple side-channel analysis: Side-channel atomicity. IEEE Trans. Comput..

[10] Liu F., Yarom Y., Ge Q., Heiser G., Lee R.B. Last-Level Cache Side-Channel Attacks Are Practical. Proceedings of the 36th IEEE Symposium on Security and Privacy.

[11] Irazoqui G., Eisenbarth T., Sunar B. S$A: A Shared Cache Attack that Works Across Cores and Defies VM Sandboxing—And Its Application to AES. Proceedings of the 36th IEEE Symposium on Security and Privacy.

[12] Oren Y., Kemerlis V.P., Sethumadhavan S., Keromytis A.D. The Spy in the Sandbox: Practical Cache Attacks in Javascript. Proceedings of the 22nd ACM SIGSAC Conference on Computer and Communications Security.

[13] Jackson C., Bortz A., Boneh D., Mitchell J.C. Protecting Browser State from Web Privacy Attacks. Proceedings of the 15th international conference on World Wide Web.

[14] Kotcher R., Pei Y., Jumde P., Jackson C. Cross-Origin Pixel Stealing: Timing Attacks Using CSS Filters. Proceedings of the 2013 ACM Conference on Computer and Communications Security.

[15] Weinberg Z., Chen E.Y., Jayaraman P.R., Jackson C. I Still Know What You Visited Last Summer: Leaking Browsing History Via User Interaction and Side Channel Attacks. Proceedings of the 2011 IEEE Symposium on Security and Privacy.

[16] Felten E.W., Schneider M.A. Timing Attacks on Web Privacy. Proceedings of the 7th ACM Conference on Computer and Communications Security.

[17] Stone P. (2013). Pixel Perfect Timing Attacks with HTML5.

[18] Jia Y., Dong X., Liang Z., Saxena P. (2015). I know where you’ve been: Geo-inference attacks via the browser cache. IEEE Internet Comput..

[19] Agrawal D., Archambeault B., Rao J.R., Rohatgi P. (2003). The EM Side-Channel(s). Proceedings of the 4th International Workshop on Cryptographic Hardware and Embedded Systems.

[20] Brier E., Joye M. (2002). Weierstraß Elliptic Curves and Side-Channel Attacks.

[21] Klein A. (2002). Cross Site Scripting Explained.

[22] Cross-Site Request Forgery. http://en.wikipedia.org/wiki/Cross-site_request_forgery.

[23] Sun F., Xu L., Su Z. (2009). Client-Side Detection of XSS Worms by Monitoring Payload Propagation. Proceedings of the 14th European Symposium on Research in Computer Security.

[24] Patil K., Dong X., Li X., Liang Z., Jiang X. Towards Fine-Grained Access Control in JavaScript Contexts. Proceedings of the International Conference on Distributed Computing Systems.

[25] Ter Louw M., Venkatakrishnan V.N. Blueprint: Robust Prevention of Cross-Site Scripting Attacks for Existing Browsers. Proceedings of the IEEE Symposium on Security and Privacy.

[26] Pokharel S., Choo K.K.R., Liu J. (2017). Mobile cloud security: An adversary model for lightweight browser security. Comput. Stand. Interfaces.

[27] Prokhorenko V., Choo K.K.R., Ashman H. (2016). Context-oriented web application protection model. Appl. Math. Comput..

[28] Prokhorenko V., Choo K.K.R., Ashman H. (2016). Intent-Based Extensible Real-Time PHP Supervision Framework. IEEE Trans. Inf. Forensics Secur..

[29] Prokhorenko V., Choo K.K.R., Ashman H. (2016). Web application protection techniques: A taxonomy. J. Netw. Comput. Appl..

[30] Chen S., Wang R., Wang X., Zhang K. Side-Channel Leaks in Web Applications: A Reality Today, a Challenge Tomorrow. Proceedings of the 2010 IEEE Symposium on Security and Privacy.

[31] Bansal C., Preibusch S., Milic-Frayling N. (2015). Cache Timing Attacks Revisited: Efficient and Repeatable Browser History, OS and Network Sniffing. ICT Systems Security and Privacy Protection.

[32] Mao J., Chen Y., Shi F., Jia Y., Liang Z. Toward Exposing Timing-based Probing Attacks in Web Applications. Proceedings of the 11th International Conference on Wireless Algorithms, Systems, and Applications (WASA).

[33] Ghinita G., Kalnis P., Tao Y. (2011). Anonymous Publication of Sensitive Transactional Data. IEEE Trans. Knowl. Data Eng..

[34] Wang Y., Cai Z., Ying G., Gao Y., Tong X., Wu G. (2016). An Incentive Mechanism with Privacy Protection in Mobile Crowdsourcing Systems. Comput. Netw..

[35] Alexa Top Sites. http://www.alexa.com/topsites.

[36] Image Scaling. https://en.wikipedia.org/wiki/Image_scaling.

[37] Bicubic Interpolation. https://en.wikipedia.org/wiki/Bicubic_interpolation.

[38] Nearest-Neighbor Interpolation. https://en.wikipedia.org/wiki/Nearest-neighbor_interpolation.

[39] Bilinear Interpolation. https://en.wikipedia.org/wiki/Bilinear_interpolation.

[40] Peak Signal-to-Noise Ratio. https://en.wikipedia.org/wiki/Peak_signal-to-noise_ratio.

